# Effect of tranexamic acid on coagulation and fibrinolysis in women with postpartum haemorrhage (WOMAN-ETAC): a single-centre, randomised, double-blind, placebo-controlled trial

**DOI:** 10.12688/wellcomeopenres.14722.1

**Published:** 2018-08-15

**Authors:** Haleema Shakur-Still, Ian Roberts, Bukola Fawole, Modupe Kuti, Oladapo O. Olayemi, Adenike Bello, Sumaya Huque, Olayinka Ogunbode, Taiwo Kotila, Chris Aimakhu, Olujide A. Okunade, Tolulase Olutogun, Cecilia O. Adetayo, Kastriot Dallaku, Ulrich Mansmann, Beverley J. Hunt, Tracey Pepple, Eni Balogun

**Affiliations:** 1 Clinical Trials Unit, London School of Hygiene and Tropical Medicine, London, WC1E 7HT, UK; 2 Department of Obstetrics & Gynaecology National Institute of Maternal and Child Health, College of Medicine, University of Ibadan, Ibadan, Nigeria; 3 Department of Chemical Pathology, College of Medicine, University of Ibadan, Ibadan, Nigeria; 4 Department of Obstetrics & Gynaecology, College of Medicine, University of Ibadan, Ibadan, Nigeria; 5 Department of Haematology, College of Medicine, University of Ibadan, Ibadan, Nigeria; 6 Institute for Medical Information Sciences, Biometry and Epidemiology, Ludwig Maximilian University of Munich, Munich, Germany; 7 University Hospital of Obstetrics Gynaecology “Koço Gliozheni”, Tirana, Albania; 8 Thrombosis & Haemophilia Centre, Guy’s & St Thomas’ Trust, London, UK

**Keywords:** Postpartum Haemorrhage, Fibrinolysis, Coagulation, Tranexamic Acid, Randomised Controlled Trial, Thromboelastogram

## Abstract

**Background:** Postpartum haemorrhage (PPH) is a leading cause of maternal death. The WOMAN trial showed that tranexamic acid (TXA) reduces death due to bleeding in women with PPH. We evaluated the effect of TXA on fibrinolysis and coagulation in a sample of WOMAN trial participants.

**Methods: **Adult women with a clinical diagnosis of PPH were randomised to receive 1 g TXA or matching placebo in the WOMAN trial. Participants in the WOMAN trial at University College Hospital (Ibadan, Nigeria) also had venous blood taken just before administration of the first dose of trial treatment and again 30 (±15) min after the first dose (the ETAC study).  We aimed to determine the effects of TXA on fibrinolysis (D-dimer and rotational thromboelastometry maximum clot lysis (ML)) and coagulation (international normalized ratio and clot amplitude at 5 min). We compared outcomes in women receiving TXA and placebo using linear regression, adjusting for baseline measurements.

**Results:** Women (n=167) were randomised to receive TXA (n=83) or matching placebo (n=84). Due to missing data, seven women were excluded from analysis. The mean (SD) D-dimer concentration was 7.1 (7.0) mg/l in TXA-treated women and 9.6 (8.6) mg/l in placebo-treated women (p=0.09). After adjusting for baseline, the D-dimer concentration was 2.16 mg/l lower in TXA-treated women (-2.16, 95% CI -4.31 to 0.00, p=0.05). There was no significant difference in ML between TXA- and placebo-treated women (12.3% (18.4) and 10.7% (12.6), respectively; p=0.52) and no significant difference after adjusting for baseline ML (1.02, 95% CI -3.72 to 5.77, p=0.67).  There were no significant effects of TXA on any other parameters.

**Conclusion:** TXA treatment was associated with reduced D-dimer levels but had no apparent effects on thromboelastometry parameters or coagulation tests.

**Registration:** ISRCTN76912190 (initially registered 10/12/2008, WOMAN-ETAC included on 22/03/2012) and NCT00872469 (initially registered 31/03/2009, WOMAN-ETAC included on 22/03/2012).

## List of abbreviations

APTT                   activated partial thromboplastin time

ETAC                   Effect of Tranexamic Acid on Coagulation

INR                      International Normalized Ratio

MCF                    maximum clot firmness 

ML                      maximum clot lysis

PPH                     postpartum haemorrhage

PT                        prothrombin Time

ROTEM               rotational thromboelastometry

SD                       standard deviation

TXA                    tranexamic acid

WOMAN Trial    World Maternal Antifibrinolytic Trial

## Introduction

Primary postpartum haemorrhage (PPH) is the leading cause of maternal death worldwide, responsible for an estimated 100,000 deaths each year ^1– 3^. Most of the deaths occur within the first 2–3 hours after giving birth and almost all (99%) are in low- and middle-income countries, with Sub-Saharan Africa accounting for the majority ^4, 5^.

Tranexamic acid (TXA) reduces bleeding by inhibiting the breakdown of fibrin and fibrinogen by the enzyme plasmin ^6^. The WOMAN trial showed that TXA reduces death due to bleeding in women with PPH by about one-fifth. When given within 3 hours of giving birth it reduces maternal death due to bleeding by around one-third ^7^. In trauma, TXA also reduces death due to bleeding by about one-third in patients treated within 3 hours of injury ^8^. However, when given after 3 hours, there is no apparent benefit ^9^. Early activation of fibrinolysis is common after trauma and is associated with increased mortality, and early administration of TXA in these patients inhibits fibrinolysis and reduces mortality ^10^. Early activation of fibrinolysis is also observed after childbirth ^11^. Within an hour of giving birth, there is a doubling of the plasma concentration of tissue plasminogen activator ^10^. Thereafter, the concentration falls rapidly ^11^. Active PPH is associated with an early increase in levels of D-dimer and plasmin-anti-plasmin complexes ^12^.

We conducted a randomised double blind placebo controlled trial (Effect of Tranexamic Acid on Coagulation, ETAC) to examine the effect of TXA on fibrinolysis and coagulation in women with PPH at the University College Hospital, Ibadan, Nigeria. We hypothesised that TXA would reduce fibrin clot breakdown (fibrinolysis) thus reducing the severity of bleeding and the risk of death.

## Methods

The aims and methods of the WOMAN ^13^ and the ETAC ^14^ trials are described in detail elsewhere. Briefly, adult women with clinically diagnosed primary PPH after vaginal or caesarean delivery were eligible Ref 7 & 13 for inclusion.

### Intervention

After the appropriate consent procedure was completed, each woman was randomly allocated to receive 1 g TXA or matching placebo by intravenous injection. If bleeding continued after 30 min, or if bleeding stopped and restarted within 24 h, a second dose of 1 g of TXA or placebo was given. We collected patient entry and outcome data as per the WOMAN trial protocol ^13^.

### Participants and blood sampling

WOMAN trial participants at University College Hospital, Ibadan, Nigeria were also considered for inclusion in the ETAC trial. In addition to the WOMAN trial procedures, women had 15 ml venous blood taken after randomisation and before administration of the first dose of trial treatment. A second venous blood sample of about 15 ml was taken 30 (±15) min after the first dose and before a second dose was given. 

### Sample analysis

We divided each venous blood sample into three vacutainer tubes. We collected one 5 ml sample in a tube containing potassium EDTA for full blood count analysis and two 4.5 ml samples in tubes containing 0.5 ml sodium citrate (0.109 mol/l) for coagulation and rotational thromboelastometry (ROTEM). We used a five-parameter particle counter Sysmex KN analyser (Sysmex Corporation, Kobe, Japan) for the full blood count analysis.

After centrifuging the blood at 3000 *g* for 20 min, we extracted the plasma without disturbing the buffy coat layer, and measured prothrombin time (PT), activated partial thromboplastin time (APTT), Clauss fibrinogen and D-dimers using a HumaClot Junior automated coagulation analyser. We measured ROTEM parameters at 37°C using two of the four channels (EXTEM, APTEM) of the ROTEM coagulation analyser (TEM®, Munich, Germany)). The ROTEM was allowed to run for 60 min. The following ROTEM variables were examined from the EXTEM and APTEM traces: clotting Time), clot amplitude at 5 and 10 min (CA-t), maximum clot firmness (MCF), maximum clot lysis (ML) and lysis index (LI). We stored the ROTEM reagents at 2–8°C with temperature monitoring and we used in-date reagents. The clinical staff had no access to the results of ROTEM analysis carried out for the ETAC trial. 

In addition, we collected the following information: time of blood samples, time the trial treatment was administered, time laboratory analysis started and ended, any treatment given that may affect coagulation, adverse events, and technical problems with analysis.

### Analyses

We published a statistical analysis plan before the allocation was unblinded ^14^. We assumed that D-dimer mean and standard deviation in the control group would be 9,000 ng/ml and 7,200 ng/ml, respectively. Taking into account that we would adjust for baseline measurement and assuming a correlation between baseline and follow-up of 0.4, we estimated that a study with about 180 patients would have 90% power (two-sided alpha=5%) to detect a reduction of 30% in the mean D-dimer value in the tranexamic group.

We conducted a per-protocol analysis that included all participants who satisfied the eligibility criteria, received the allocated treatment, had follow up samples and at least one measurement of the primary outcome. We did not exclude outliers or impute missing data as it would be inappropriate in a study aimed at understanding the biological effects of TXA.

### Outcomes

We assessed the effect of TXA on fibrinolysis by assessing D-dimer and ML as our co-primary outcome. For each co-primary outcome, we compared the follow up results of each treatment group (t-test). 

Our secondary outcomes included INR, PT, APTT, fibrinogen, haemoglobin and the following ROTEM EXTEM parameters: clotting time (CT), clot amplitude at 5 (A5) and 10 min (A10), LI at 30 and 60 min and MCF. We compared the mean follow-up result in women receiving TXA and those receiving placebo using the t-test. We also compared follow-up results of each treatment group using linear regression, adjusting for the corresponding baseline measurement.

### Statistical analysis

The effect of TXA on our co-primary outcomes, D-dimer and maximum lysis, were explored stratified by time since delivery, type of delivery, cause of postpartum haemorrhage and maternal anaemia. A t-test was conducted to compare means between treatment arms, and the likelihood ratio test was used to test for interaction between subgroups. We defined hyperfibrinolysis as ML>15% on ROTEM EXTEM and coagulopathy as an INR >1.2 and A5 ≤40 mm. We used logistic regression to assess multivariate odds ratios between baseline variables and hyperfibrinolysis and coagulopathy. Stata 15 was used for all statistical analyses ^15^.

To set the results of the ETAC study in context of an almost identical haematological sub-study conducted within the WOMAN trial but in a different location (Albania) and provide a more robust estimate of the effect of TXA on D-dimer, we conducted a meta-analysis of the two studies. Data from the ETAC trial and ETAPlaT (a single centre sub-study of the WOMAN trial) were pooled. Eligibility criteria for both trials were the same and blood samples were collected for D-dimer in the same way in both trials ^16^. We computed the pooled ratio of D-dimers in women receiving TXA compared to women receiving placebo. We log-transformed individual patient data for D-dimer, and calculated the arithmetic mean (SD) of the log-transformed values for each study. A meta- analysis of the arithmetic means of transformed data gives a mean difference, which after back-transforming, corresponds to a meta-analysis of the ratio of geometric means in the original scale. Statistical heterogeneity was examined by visual inspection of forest plots, the I ^2^ statistic and χ ^2^ test. This analysis was not included in the statistical analysis plan for the ETAC trial.

### Ethical approval and consent to participate

The trial was conducted in accordance with the ICH-GCP ^17^. Approvals were obtained from the Ethics Committees of London school of Hygiene and Tropical Medicine (Reference A275 5536) and the University of Ibadan & University College Hospital Ethics Committee (Reference UI/EC/09/0131). Regulatory approval was obtained from the Nigerian National Agency for Food and Drug Administration and Control.

The consent procedures are described in detail in the WOMAN Trial protocol ^13^. In summary, consent was obtained from women if their physical and mental capacity allowed. If a woman was unable to give consent, proxy consent was obtained from a relative or representative. If a proxy was unavailable, then consent was deferred or waived. When consent was deferred or given by a proxy, the woman was informed about the trial as soon as possible, and consent obtained for on-going data collection, if needed.

## Results

### Participation

**Figure 1.**
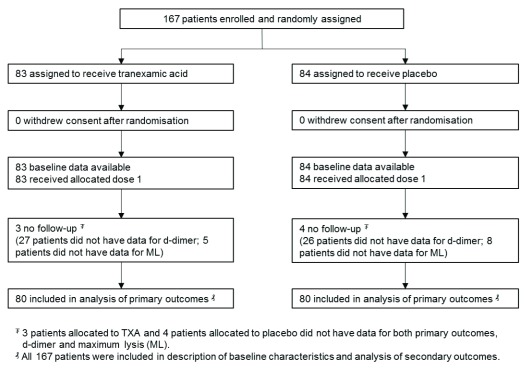
Consort diagram.

The ETAC trial recruited patients from April 2012 to March 2016. ** A total of 205 women were recruited into the WOMAN trial at University College Hospital, Ibadan during this period of whom 167 were included in the ETAC trial. The planned sample size of 180 participants was not reached due to occasional equipment failures and interruptions in the supply of reagents. Participants were randomly assigned to receive TXA (n=83) or placebo (n=84). All participants received the first dose of the allocated treatment, and no one withdrew their consent. Data on both co-primary outcomes were missing for 7 women, and they were excluded from the primary analysis. The CONSORT flow diagram is shown in Figure 1. The dataset generated for this study is available online following registration ^18^.

### Participant characteristics

Table 1 reports baseline characteristics of trial participants by treatment group. A total of 128 (77%) gave birth in the hospital, whereas 39 (23%) gave birth in other settings and were admitted to hospital after PPH onset. Age and systolic blood pressure were normally distributed; all other continuous variables had a skewed distribution. Therefore, we present means, medians and interquartile ranges for all variables. The mean ROTEM CT at baseline was higher in women randomised to TXA (180 s) compared to placebo (78 s). All other baseline parameters were similar between the two treatment arms.

**Table 1.** Baseline characteristics by treatment group (N=167).

	TXA (N = 83)			Placebo (N = 84)		
	n ^a^	Mean (SD)/ count (%)	Median (IQR)	n ^a^	Mean (SD)/ Count (%)	Median (IQR)
Age	83	32.1 (5.8)	32 (29, 36)	84	31.6 (5.4)	31 (28, 35)
>35 years		28 (33.7%)			21 (25.0%)	
Time since delivery	83	4.5 (6.2)	1.7 (0.8, 6.3)	84	3.9 (4.5)	1.9 (0.9, 5.0)
≤3 hours		52 (62.7%)			51 (60.7%)	
>3 hours		31 (37.3%)			33 (39.3%)	
Type of delivery	83			84		
Vaginal		37 (44.6%)			43 (51.2%)	
Caesarean section		46 (55.4%)			41 (48.8%)	
Delivery in randomising hospital	83			84		
Yes		62 (74.7%)			66 (78.6%)	
No		21 (25.3%)			18 (21.4%)	
Cause of Haemorrhage	83			84		
Uterine Atony		41 (49.4%)			33 (39.3%)	
Other		42 (50.6%)			51 (60.7%)	
Systolic blood pressure (mmHg)	83	108.2 (26.7)	110 (90, 120)	83	111.7 (27.4)	110 (90, 130)
<90 mmHg		17 (20.5%)			14 (16.9%)	
Blood loss volume	83	1638.9 (1016.9)	1300 (1000, 2000)	84	1423.8 (751.7)	1200 (825, 1950)
>1000 ml		56 (67.5%)			52 (61.9%)	
Clinical signs of shock	83			84		
Yes		54 (65.1%)			51 (60.7%)	
No		29 (34.9%)			33 (39.3%)	
Full Blood Count Variables						
Haemoglobin (g/l)	65	72.6 (32.6)	72 (55, 95)	67	83.4 (37.8)	84 (63, 100)
<110 g/l		59 (90.8%)			57 (85.1%)	
White cell count (x10 ^9^/l)	66	12.2 (7.6)	11.5 (6, 17.3)	65	11.7 (8.7)	10.7 (5.3, 15.4)
Platelet count (x10 ^9^/l)	65	151.6 (114.9)	126 (84, 203)	65	158.9 (87.2)	161 (86, 212)
Coagulation Variables						
INR	67	1.6 (1.4)	1.2 (1, 1.6)	70	1.4 (0.8)	1.1 (1.0, 1.3)
INR>1.2		33 (49.3%)			24 (34.3%)	
PT (s)	67	20.5 (15.6)	15.8 (13.5, 19.8)	70	17.6 (8.6)	14.8 (13.5, 16.8)
APTT (s)	64	36.4 (24.7)	29.9 (26.8, 37.0)	69	35.4 (21.3)	30.7 (27.1, 33.5)
Fibrinogen (g/l)	66	8.9 (7.1)	7.2 (3.2, 12.7)	70	8.0 (6.1)	6.0 (3.3, 11.9)
D-dimer (mg/l)	59	7.7 (8.6)	4.3 (1.5, 11.1)	60	9.5 (7.7)	6.8 (3.4, 15.2)
Thromboelastometry (ROTEM® EXTEM) variables						
CT (seconds)	73	179.9 (469.9)	54 (45, 82)	78	78.3 (97.5)	54.5 (45, 68)
ML (%)	72	17.0 (25.0)	9 (4, 18)	78	12.6 (14.9)	9 (4, 14)
ML>15%		20 (27.8%)			15 (19.2%)	
A5 (mm)	69	39.4 (18.0)	44 (29, 52)	77	44.6 (13.2)	48 (38, 53)
A5<40		28 (40.6%)			21 (27.3%)	
A10 (mm)	72	48.2 (20.5)	55 (39, 63)	77	54.7 (13.6)	59 (51, 63)
LI30 (%)	68	96.5 (15.6)	100 (100, 100)	77	99.3 (1.8)	100 (100, 100)
LI60 (%)	46	93.7 (11.6)	97.5 (92, 99)	55	93.0 (8.7)	95 (91, 99)
MCF (mm)	69	56.1 (20.6)	64 (51, 70)	77	62.0 (13.6)	65 (61, 69)

^a^Number of women with available data. ^b^Two women in the tranexamic acid arm had outlying ROTEM EXTEM clotting time values of 1814 and 3468 seconds. TXA, tranexamic acid; INR, international normalized ratio; PT, prothrombin time; APTT, activated partial prothromboplastin time; CT, clotting time; ML, maximum lysis; A5, clot amplitude at 5 min; LI30, lysis index at 30 min; MCF, maximum clot firmness.

Follow-up venous blood was collected at 31 (6) (mean (SD)) minutes after administration of TXA or matching placebo and all samples were obtained before a second dose was given. The mean (SD) time between treatment and collection of the follow up blood sample was 30 (4) min in the TXA arm and 33 (8) minutes in the placebo arm. 

The effect of TXA on fibrinolysis and coagulation is reported in Table 2. The mean (SD) D-dimer level after treatment was 7.1 (7.0) mg/l in women receiving TXA and 9.6 (8.6) in women receiving the placebo. After adjusting for baseline D-dimer level, the difference in mean D-dimer between women receiving TXA and placebo was -2.16 (95% CI: -4.31 to 0.00, p=0.05). There was no significant difference in ML between TXA- and placebo-treated women (12.3% (18.4) and 10.7% (12.6), respectively; p = 0.52) and no significant difference after adjusting for baseline ML (1.02, 95% CI -3.72 to 5.77, p= 0.67). There were no significant effects of TXA on any other parameters. There were no adverse events associated with this sub-study reported. 

**Table 2.** Effect of tranexamic acid (TXA) on coagulation and fibrinolysis in women with postpartum haemorrhage.

	n	TXA	n	Placebo	Difference (95% CI)	p-value †	Baseline adjusted difference (95% CI) *	p-value ‡
		Mean (SD)		Mean (SD)				
**Primary outcomes**								
D-dimer (mg/l)	56	7.1 (7.0)	58	9.6 (8.6)	-2.5 (-5.4, 0.4)	0.09	-2.2 (-4.3, 0.0)	0.05
ML (%)	78	12.3 (18.4)	76	10.7 (12.6)	1.6 (-3.4, 6.7)	0.52	1.0 (-3.7, 5.8)	0.67
**Secondary outcomes**								
INR	71	1.6 (1.8)	70	1.2 (0.4)	0.4 (-0.1, 0.8)	0.09	0.4 (-0.1, 0.9)	0.11
PT (s)	71	18.5 (15.6)	70	15.8 (4.7)	2.7 (-1.1, 6.5)	0.17	2.5 (-1.6, 6.6)	0.22
APTT (s)	71	36.3 (20.7)	69	33.2 (12.6)	3.0 (-2.7, 8.8)	0.30	1.0 (-3.9, 5.8)	0.69
Fibrinogen (g/l)	68	8.8 (6.1)	69	8.8 (6.5)	-0.03 (-2.2, 2.1)	0.98	-1.1 (-2.3, 0.2)	0.08
Haemoglobin (g/l)	71	75.4 (30.9)	67	83.2 (31.1)	-7.8 (-18.3, 2.6)	0.14	-1.0 (-8.8, 6.8)	0.79
**Thromboelastometry (ROTEM® EXTEM)**								
CT (s)	78	151.3 (528.4)	77	104.7 (385.9)	46.6 (-100.4, 193.6)	0.53	-8.8 (-150.1, 132.4)	0.90
A5 (mm)	75	39.5 (16.7)	76	45.3 (12.9)	-5.8 (-10.6, -1.0)	0.02	-2.0 (-5.7, 1.7)	0.27
A10 (mm)	78	49.6 (18.1)	76	55.3 (13.1)	-5.7 (-10.8, -0.7)	0.03	-1.7 (-5.9, 2.5)	0.41
LI30 (%)	71	98.3 (11.3)	75	99.2 (3.6)	-0.8 (-3.5, 1.9)	0.54	-0.9 (-3.9, 2.0)	0.52
LI60 (%)	49	93.6 (13.0)	49	93.3 (11.5)	0.2 (-4.7, 5.2)	0.92	-0.3 (-5.6, 4.9)	0.90
MCF (mm)	72	57.4 (18.1)	70	62.6 (11.1)	-5.2 (-10.2, -0.2)	0.04	-1.8 (-6.2, 2.5)	0.40

†p-value from t-test. *Adjusted for the corresponding baseline parameter e.g. effect of TXA on D-dimer adjusted for pre-treatment D-dimer values. ‡p-value from likelihood ratio test (linear regression). INR, international normalized ratio; PT, prothrombin time; APTT, activated partial prothromboplastin time; CT, clotting time; A5, clot amplitude at 5 min; LI30, lysis index at 30 min; MCF, maximum clot firmness.

There was no evidence of heterogeneity in the effect of TXA on fibrinolysis (D-dimer and ML) by time since delivery, type of delivery, cause of haemorrhage and severity of anaemia.

## Discussion

**Figure 2.**
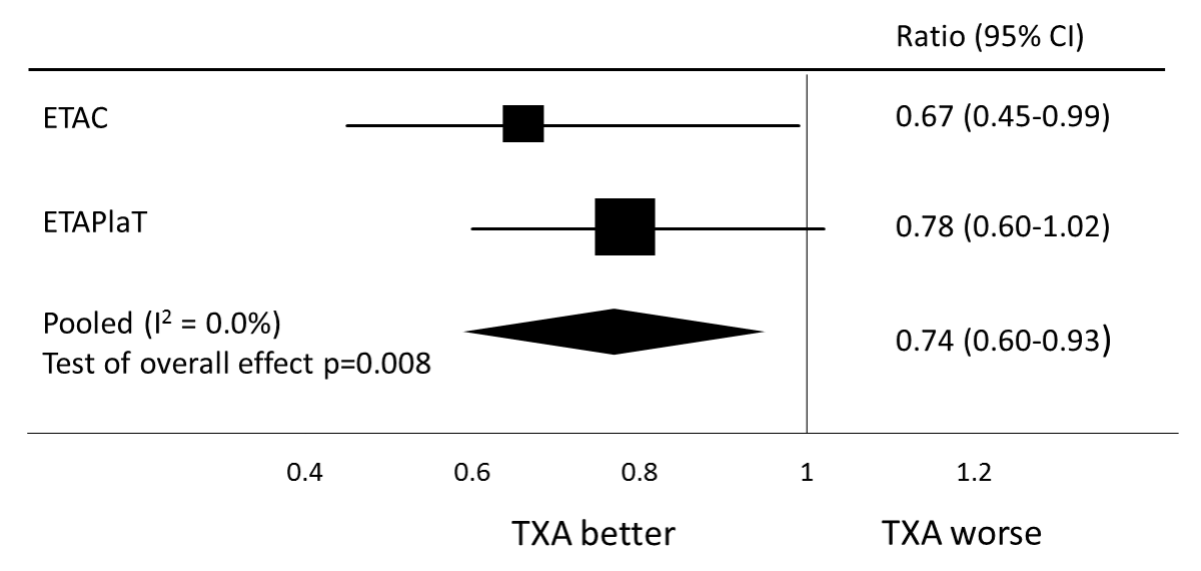
Forest plot of the effect of tranexamic acid (TXA) on D-dimer values in women with postpartum haemorrhage.

TXA treatment was associated with reduced D-dimer levels, but had no apparent effects on ROTEM parameters or coagulation tests. The effect of TXA on D-dimer levels in this study is similar to that observed in an almost identical WOMAN trial sub-study that aimed to assess the effects of TXA on platelet function (ETAPLAT-study) ^16^. The eligibility criteria for both studies were the same and blood samples for D-dimer were collected in the same way. When the results of the two sub-studies are pooled in a meta-analysis ( Figure 2), there is a 26% reduction in D-dimer levels with TXA (pooled D-dimer ratio 0.74, 95% CI 0.60 to 0.93, p=0.008). These results concur with a study in France that showed the early increase in D-dimer values in women with PPH can be inhibited by TXA ^11^.

Although we planned to include 180 women in our study, due to equipment and power failures, and interruptions in the supply of reagents, only 167 women were recruited. Power cuts are common in Nigeria and although back-up power packs were supplied with the ROTEM delta analyser, these also failed on occasions. ROTEM reagents have a short shelf-life and were sourced in Europe since they are not readily available in Nigeria. Moreover, due to technical problems with blood samples or measurement instruments, the number of women with useable outcome data was less than the number of women enrolled. As a result, the power of the study to detect differences between treatment arms was lower than anticipated. This lack of power may at least partially explain the absence of any significant effects of TXA on thromboelastometry parameters and coagulation tests.

Increased fibrinolysis is common in women with PPH ^19^. Our results show that this increase can be inhibited with TXA. Larger studies into the effects of TXA on fibrinolysis and coagulation in women with or at risk from PPH are required.

## Data availability

The anonymised trial data is available from the freeBIRD data portal at https://ctu-app.lshtm.ac.uk/freebird/index.php/data-sharing/downloads/woman-etac/ following free registration: http://dx.doi.org/10.17037/DATA.00000788 ^18^. Data are available under an Open Data Commons Attribution License (ODC-By) licence.

The trial protocol, statistical analysis plan and trial publications will be made freely available at http://www.txacentral.org/ and http://womantrial.lshtm.ac.uk/

## Supplementary information

**Supplementary File 1. Completed CONSORT checklist.**
