# Supplementary material for: Effect of tranexamic acid on coagulation and fibrinolysis in women with postpartum haemorrhage (WOMAN-ETAC): a single-centre, randomised, double-blind, placebo-controlled trial
Source: Wellcome Open Res. 2018 Aug 15;3:100. [Version 1] doi: 10.12688/wellcomeopenres.14722.1 (PMC6171555; doi:10.12688/wellcomeopenres.14722.1)
Supplement: Peer review history

# Peer review: Referee response for version 1

**DOI:** 10.21956/wellcomeopenres.16038.r33855

**Published:** 2018-09-24

**Authors:** Stanislas Grassin-Delyle

**Affiliations:** 1 Foch Hospital, Respiratory Diseases Department, Versailles Saint-Quentin-en-Yvelines University, University of Paris-Saclay, Suresnes, France

Shakur-Still and colleagues investigated the effect of tranexamic acid on fibrinolysis and coagulation parameters in women with primary postpartum haemorrhage. The study was a randomised double-blind placebo-controlled trial and the inclusion criteria were the same as in the WOMAN clinical trial previously published by this team. Female participants were randomized to receive 1g intravenous tranexamic acid or placebo, and blood samples were taken before administration and 30 minutes later to assess coagulation and fibrinolysis parameters. On these samples, the results show a lower D-dimer concentration in the treated group than in the placebo group, after correction of the baseline concentration. These results are confirmed by the presentation of a meta-analysis including the results of a similar study conducted in another centre. This work is remarkable for the implementation of specialized methods of off-site biology under non-ideal conditions. The study is very well conducted and all methods and results are clearly reported. This study provides additional evidence on the action of tranexamic acid on fibrinolysis, which is the main mechanism involved in reducing bleeding and mortality. This work therefore further strengthens the interest in tranexamic acid for the treatment of haemorrhagic diseases, even if its pharmacology remains poorly known, in particular the optimal dose and administration method.

 One of the technical aspects that could be clarified concerns the taking of blood samples. Each 15 ml sample was divided into three fractions: one 5 ml sample in a tube containing potassium EDTA for full blood count analysis and two 4.5 ml samples in tubes containing 0.5 ml sodium citrate for coagulation and rotational thromboelastometry. It would be interesting to know the nature of the primary tube used for sampling and the time frame within which the distribution between the three tubes was then carried out, in order to be able to assess the risk of initial coagulation activation.

I have read this submission. I believe that I have an appropriate level of expertise to confirm that it is of an acceptable scientific standard.

# Peer review: Referee response for version 1

**DOI:** 10.21956/wellcomeopenres.16038.r33856

**Published:** 2018-09-13

**Authors:** Homa K. Ahmadzia

**Affiliations:** 1 Division of Maternal-Fetal Medicine, Department of Obstetrics and Gynecology, School of Medicine and Health Sciences, George Washington University , Washington, DC, USA

Summary: This article by Shakur-Still et al. studies the coagulation markers affected by tranexamic acid administration to women with clinically diagnosed postpartum hemorrhage using traditional and newer methods of laboratory analysis. This is a timely study as very limited data exists what happens in women with actual diagnosed postpartum hemorrhage and their coagulation parameters with and without antifibrinolytic therapy.

 Introduction:

 While it is true that coagulation parameters are affected after childbirth, as described, the wrong citation is provided for the statement 'within an hour of giving birth, there is a doubling of the plasma concentration of tissue plasminogen activator'.

 Statistical analysis:

 Would be prudent to provide the citation/rationale for using ML>15% as 'hyperfibrinolysis' as well as the other parameters/values cited.

 Results:

 Would be good to cite original trial methods or briefly describe randomization scheme employed for who got TXA versus placebo.

 Table 1-Please provide statistical statement specifically how baseline parameters were similar (i.e. p>0.05) either in text or footnote other than the baseline CT values.

 When making the statement 'no evidence of heterogeneity in the effect of TXA on fibrinolysis by time since delivery, etc' please provide actual data/analysis to support this finding. 

 Although the authors state no outlier was excluded, it may be worth examining in a subgroup analysis or sensitivity analysis what the ROTEM parameter differences would be if the outlier groups where not included - for example in the footnote in table 1 it states CT values for two women were 1814 and 3468 seconds. This either means the test was run in error by the user or that the coagulopathy makes it difficult to interpret ML parameter.

 Also, where these two women (from Table 1 in the footnote) included for the final comparison for ML?

 Also one other key question is if the CONSORT flow chart states 80 women were included for primary outcome analysis - why are only 56/58 women shown data for d-dimer values in Table 2? Also it is interesting that only 49 patients per group were included for the LI60%. Were there technical issues that made it not possible to report out the LI60% parameter - if so then that could mean the ML parameter may also be less reliable.

 If these are correct interpretations, please include in limitations section of discussion.

I have read this submission. I believe that I have an appropriate level of expertise to confirm that it is of an acceptable scientific standard.
